# Supplementary material for: Meta-Analysis and Gene Set Enrichment Relative to ER Status Reveal Elevated Activity of MYC and E2F in the “Basal” Breast Cancer Subgroup
Source: PLoS One. 2009 Mar 9;4(3):e4710. doi: 10.1371/journal.pone.0004710 (PMC2650420; doi:10.1371/journal.pone.0004710)
Supplement: Dataset S1 — GSEA results from first screen. The file contains a zipped archive of the HTML results of the second GSEA screen. Reports for enriched or depleted datasets may be accessed from the “index.html” link within the my_analysis.GseaPreranked.1219975950625.rpt.zip ZIP archive. Enrichment plots are available for each of the 50 most enriched and depleted gene sets. (13.32 MB ZIP) [file pone.0004710.s001.zip › my_analysis.GseaPreranked.1219975950625.rpt.zip!//V$E2F1_Q6.html]

Details for gene set V$E2F1\_Q6[GSEA]

|  || Dataset | zSco.ERneg.to.ERpos\_collapsed |
| Phenotype | NoPhenotypeAvailable |
| Upregulated in class | na\_pos |
| GeneSet | V$E2F1\_Q6 |
| Enrichment Score (ES) | 0.4966577 |
| Normalized Enrichment Score (NES) | 2.1954255 |
| Nominal p-value | 0.0 |
| FDR q-value | 0.0 |
| FWER p-Value | 0.0 |
Table: GSEA Results Summary

  

Fig 1: Enrichment plot: V$E2F1\_Q6      
 Profile of the Running ES Score & Positions of GeneSet Members on the Rank Ordered List

  

| PROBE | GENE SYMBOL | GENE\_TITLE | RANK IN GENE LIST | RANK METRIC SCORE | RUNNING ES | CORE ENRICHMENT || 1 | E2F3 |  |  | 6 | 7.369 | 0.0201 | Yes |
| 2 | NASP |  |  | 59 | 6.201 | 0.0335 | Yes |
| 3 | PIM1 |  |  | 78 | 6.025 | 0.0489 | Yes |
| 4 | AK2 |  |  | 85 | 5.971 | 0.0651 | Yes |
| 5 | KLF5 |  |  | 87 | 5.966 | 0.0817 | Yes |
| 6 | GPRC5B |  |  | 103 | 5.845 | 0.0969 | Yes |
| 7 | MCM4 |  |  | 180 | 5.436 | 0.1062 | Yes |
| 8 | MCM6 |  |  | 198 | 5.317 | 0.1198 | Yes |
| 9 | GMNN |  |  | 206 | 5.302 | 0.1341 | Yes |
| 10 | ORC1L |  |  | 212 | 5.280 | 0.1484 | Yes |
| 11 | PAX6 |  |  | 224 | 5.208 | 0.1621 | Yes |
| 12 | HMGA1 |  |  | 244 | 5.125 | 0.1750 | Yes |
| 13 | CDC25A |  |  | 281 | 4.993 | 0.1862 | Yes |
| 14 | MCM7 |  |  | 289 | 4.970 | 0.1995 | Yes |
| 15 | STMN1 |  |  | 310 | 4.907 | 0.2117 | Yes |
| 16 | EZH2 |  |  | 312 | 4.888 | 0.2252 | Yes |
| 17 | ARHGAP11A |  |  | 361 | 4.763 | 0.2349 | Yes |
| 18 | RANBP1 |  |  | 380 | 4.733 | 0.2467 | Yes |
| 19 | SYNCRIP |  |  | 402 | 4.679 | 0.2582 | Yes |
| 20 | SNRPD1 |  |  | 440 | 4.552 | 0.2681 | Yes |
| 21 | DMD |  |  | 481 | 4.406 | 0.2773 | Yes |
| 22 | SUV39H1 |  |  | 495 | 4.361 | 0.2885 | Yes |
| 23 | TYRO3 |  |  | 516 | 4.316 | 0.2990 | Yes |
| 24 | RPS20 |  |  | 615 | 4.101 | 0.3030 | Yes |
| 25 | MCM2 |  |  | 630 | 4.080 | 0.3134 | Yes |
| 26 | MCM3 |  |  | 656 | 4.019 | 0.3227 | Yes |
| 27 | NCL |  |  | 678 | 3.977 | 0.3322 | Yes |
| 28 | TMPO |  |  | 792 | 3.788 | 0.3342 | Yes |
| 29 | ILF3 |  |  | 801 | 3.774 | 0.3441 | Yes |
| 30 | MSH2 |  |  | 819 | 3.748 | 0.3533 | Yes |
| 31 | MYC |  |  | 1025 | 3.447 | 0.3473 | Yes |
| 32 | UNG |  |  | 1070 | 3.390 | 0.3534 | Yes |
| 33 | FANCC |  |  | 1175 | 3.264 | 0.3546 | Yes |
| 34 | NOLC1 |  |  | 1222 | 3.217 | 0.3601 | Yes |
| 35 | PRKDC |  |  | 1234 | 3.204 | 0.3682 | Yes |
| 36 | DNAJC9 |  |  | 1243 | 3.198 | 0.3765 | Yes |
| 37 | CDC2 |  |  | 1259 | 3.182 | 0.3843 | Yes |
| 38 | POLD1 |  |  | 1260 | 3.182 | 0.3932 | Yes |
| 39 | CDC5L |  |  | 1278 | 3.162 | 0.4007 | Yes |
| 40 | FBXO5 |  |  | 1304 | 3.137 | 0.4075 | Yes |
| 41 | SFRS10 |  |  | 1320 | 3.118 | 0.4151 | Yes |
| 42 | PCNA |  |  | 1346 | 3.087 | 0.4218 | Yes |
| 43 | ING3 |  |  | 1398 | 3.042 | 0.4264 | Yes |
| 44 | EFNA5 |  |  | 1488 | 2.953 | 0.4279 | Yes |
| 45 | ATAD2 |  |  | 1532 | 2.917 | 0.4328 | Yes |
| 46 | PRPS2 |  |  | 1592 | 2.859 | 0.4363 | Yes |
| 47 | EPHB1 |  |  | 1675 | 2.780 | 0.4378 | Yes |
| 48 | NUP62 |  |  | 1684 | 2.770 | 0.4449 | Yes |
| 49 | E2F1 |  |  | 1695 | 2.764 | 0.4519 | Yes |
| 50 | HIRA |  |  | 1696 | 2.763 | 0.4596 | Yes |
| 51 | PKMYT1 |  |  | 1715 | 2.753 | 0.4659 | Yes |
| 52 | FKBP5 |  |  | 1729 | 2.743 | 0.4726 | Yes |
| 53 | SUMO1 |  |  | 1736 | 2.736 | 0.4798 | Yes |
| 54 | UGCGL1 |  |  | 1779 | 2.709 | 0.4841 | Yes |
| 55 | CASP8AP2 |  |  | 1930 | 2.587 | 0.4800 | Yes |
| 56 | RRM2 |  |  | 2047 | 2.485 | 0.4781 | Yes |
| 57 | H2AFZ |  |  | 2105 | 2.447 | 0.4806 | Yes |
| 58 | MAP3K7 |  |  | 2143 | 2.421 | 0.4845 | Yes |
| 59 | SFRS7 |  |  | 2226 | 2.370 | 0.4849 | Yes |
| 60 | POLR2A |  |  | 2274 | 2.334 | 0.4878 | Yes |
| 61 | FANCG |  |  | 2282 | 2.332 | 0.4938 | Yes |
| 62 | SOAT1 |  |  | 2362 | 2.281 | 0.4942 | Yes |
| 63 | IPO7 |  |  | 2413 | 2.247 | 0.4967 | Yes |
| 64 | CDT1 |  |  | 2522 | 2.190 | 0.4946 | No |
| 65 | HNRPD |  |  | 2585 | 2.156 | 0.4959 | No |
| 66 | DNMT1 |  |  | 2844 | 2.017 | 0.4819 | No |
| 67 | ATF5 |  |  | 2941 | 1.962 | 0.4801 | No |
| 68 | RASAL2 |  |  | 3067 | 1.901 | 0.4759 | No |
| 69 | SSBP3 |  |  | 3087 | 1.891 | 0.4797 | No |
| 70 | KPNB1 |  |  | 3125 | 1.876 | 0.4822 | No |
| 71 | CDC6 |  |  | 3185 | 1.847 | 0.4828 | No |
| 72 | C10ORF119 |  |  | 3202 | 1.838 | 0.4867 | No |
| 73 | PAQR4 |  |  | 3434 | 1.736 | 0.4740 | No |
| 74 | PRPF4B |  |  | 3435 | 1.736 | 0.4789 | No |
| 75 | SMAD6 |  |  | 3436 | 1.736 | 0.4837 | No |
| 76 | TOPBP1 |  |  | 3475 | 1.722 | 0.4856 | No |
| 77 | SLCO3A1 |  |  | 3563 | 1.679 | 0.4837 | No |
| 78 | MSH5 |  |  | 3656 | 1.639 | 0.4813 | No |
| 79 | SLC9A7 |  |  | 3790 | 1.589 | 0.4756 | No |
| 80 | PRPS1 |  |  | 3889 | 1.556 | 0.4725 | No |
| 81 | POLE2 |  |  | 3975 | 1.520 | 0.4703 | No |
| 82 | STAG2 |  |  | 4224 | 1.421 | 0.4555 | No |
| 83 | RQCD1 |  |  | 4253 | 1.412 | 0.4573 | No |
| 84 | HNRPUL1 |  |  | 4310 | 1.387 | 0.4569 | No |
| 85 | GRIA4 |  |  | 4341 | 1.377 | 0.4585 | No |
| 86 | CLSPN |  |  | 4483 | 1.324 | 0.4514 | No |
| 87 | GABRB3 |  |  | 4549 | 1.303 | 0.4501 | No |
| 88 | CACNA1G |  |  | 4739 | 1.239 | 0.4392 | No |
| 89 | KIAA0101 |  |  | 4770 | 1.230 | 0.4404 | No |
| 90 | STAG1 |  |  | 4794 | 1.225 | 0.4421 | No |
| 91 | CORT |  |  | 4877 | 1.196 | 0.4392 | No |
| 92 | HNRPR |  |  | 4913 | 1.185 | 0.4398 | No |
| 93 | HTF9C |  |  | 4986 | 1.161 | 0.4376 | No |
| 94 | ARHGAP6 |  |  | 4991 | 1.159 | 0.4405 | No |
| 95 | MYH10 |  |  | 5076 | 1.124 | 0.4373 | No |
| 96 | FLI1 |  |  | 5103 | 1.114 | 0.4384 | No |
| 97 | HIST1H4A |  |  | 5241 | 1.067 | 0.4310 | No |
| 98 | EED |  |  | 5267 | 1.060 | 0.4320 | No |
| 99 | CCNT1 |  |  | 5647 | 0.931 | 0.4059 | No |
| 100 | ACO2 |  |  | 5713 | 0.907 | 0.4034 | No |
| 101 | SEMA6A |  |  | 5935 | 0.836 | 0.3890 | No |
| 102 | PB1 |  |  | 5977 | 0.822 | 0.3882 | No |
| 103 | ADAMTS2 |  |  | 5988 | 0.817 | 0.3897 | No |
| 104 | YBX2 |  |  | 6299 | 0.716 | 0.3681 | No |
| 105 | ALDH6A1 |  |  | 6317 | 0.711 | 0.3688 | No |
| 106 | H2AFV |  |  | 6569 | 0.627 | 0.3515 | No |
| 107 | SPTB |  |  | 6641 | 0.609 | 0.3478 | No |
| 108 | POLD3 |  |  | 6667 | 0.601 | 0.3476 | No |
| 109 | TFAP4 |  |  | 6777 | 0.561 | 0.3409 | No |
| 110 | RBBP4 |  |  | 7160 | 0.432 | 0.3131 | No |
| 111 | AP1S1 |  |  | 7316 | 0.377 | 0.3024 | No |
| 112 | GATA1 |  |  | 7431 | 0.334 | 0.2946 | No |
| 113 | TBX6 |  |  | 7723 | 0.223 | 0.2732 | No |
| 114 | SP3 |  |  | 7914 | 0.141 | 0.2591 | No |
| 115 | PVRL1 |  |  | 8019 | 0.098 | 0.2515 | No |
| 116 | SLC9A5 |  |  | 8303 | 0.000 | 0.2300 | No |
| 117 | MXD3 |  |  | 8312 | -0.002 | 0.2294 | No |
| 118 | HIST1H2BK |  |  | 8356 | -0.020 | 0.2262 | No |
| 119 | HMG2L1 |  |  | 8412 | -0.042 | 0.2221 | No |
| 120 | SYNGR4 |  |  | 8638 | -0.147 | 0.2054 | No |
| 121 | FMO4 |  |  | 8775 | -0.199 | 0.1957 | No |
| 122 | SFRS1 |  |  | 8891 | -0.255 | 0.1877 | No |
| 123 | DCK |  |  | 8935 | -0.280 | 0.1852 | No |
| 124 | ID3 |  |  | 8993 | -0.308 | 0.1817 | No |
| 125 | PPM1D |  |  | 9078 | -0.350 | 0.1763 | No |
| 126 | SLC25A3 |  |  | 9309 | -0.459 | 0.1601 | No |
| 127 | FLJ25476 |  |  | 9347 | -0.481 | 0.1586 | No |
| 128 | PCSK1 |  |  | 9351 | -0.483 | 0.1598 | No |
| 129 | MAZ |  |  | 9415 | -0.521 | 0.1564 | No |
| 130 | HIST1H3A |  |  | 9503 | -0.563 | 0.1514 | No |
| 131 | ZCCHC8 |  |  | 9511 | -0.566 | 0.1524 | No |
| 132 | HNRPA2B1 |  |  | 9531 | -0.578 | 0.1526 | No |
| 133 | ASXL2 |  |  | 9736 | -0.689 | 0.1390 | No |
| 134 | ZCWPW1 |  |  | 9773 | -0.709 | 0.1383 | No |
| 135 | GSPT1 |  |  | 9841 | -0.751 | 0.1353 | No |
| 136 | SALL1 |  |  | 9959 | -0.818 | 0.1287 | No |
| 137 | MRPL40 |  |  | 10273 | -1.004 | 0.1077 | No |
| 138 | CTNND2 |  |  | 10581 | -1.227 | 0.0878 | No |
| 139 | XTP3TPA |  |  | 10592 | -1.236 | 0.0905 | No |
| 140 | C11ORF30 |  |  | 10656 | -1.284 | 0.0893 | No |
| 141 | C14ORF130 |  |  | 10945 | -1.523 | 0.0717 | No |
| 142 | AP4M1 |  |  | 11123 | -1.683 | 0.0629 | No |
| 143 | CTCF |  |  | 11128 | -1.687 | 0.0673 | No |
| 144 | CSPG2 |  |  | 11207 | -1.748 | 0.0663 | No |
| 145 | PHF15 |  |  | 11215 | -1.755 | 0.0707 | No |
| 146 | HOXC10 |  |  | 11345 | -1.878 | 0.0661 | No |
| 147 | FHOD1 |  |  | 11408 | -1.941 | 0.0668 | No |
| 148 | USP52 |  |  | 11931 | -2.478 | 0.0341 | No |
| 149 | CXORF12 |  |  | 12144 | -2.762 | 0.0257 | No |
| 150 | RPS6KA5 |  |  | 12285 | -2.949 | 0.0233 | No |
| 151 | NELL2 |  |  | 12593 | -3.420 | 0.0095 | No |
| 152 | SFRS12 |  |  | 12641 | -3.513 | 0.0157 | No |
| 153 | ARID4A |  |  | 12806 | -3.906 | 0.0142 | No |
| 154 | ERBB2IP |  |  | 12910 | -4.180 | 0.0180 | No |
| 155 | APPL |  |  | 13059 | -4.678 | 0.0198 | No |
Table: GSEA details [plain text format]

  

Fig 2: V$E2F1\_Q6: Random ES distribution      
 Gene set null distribution of ES for **V$E2F1\_Q6**
